# ASO Author Reflections: Smoking Status Impact on Perioperative Morbidity and Long-Term Survival of Patients Undergoing Esophagectomy for Cancer

**DOI:** 10.1245/s10434-021-09765-7

**Published:** 2021-03-05

**Authors:** Sivesh K. Kamarajah, Alexander W. Phillips

**Affiliations:** 1 Northern Oesophagogastric Unit, Royal Victoria Infirmary, Newcastle University Trust Hospitals, Newcastle-Upon-Tyne, UK; 2 School of Medical Education, Newcastle University, Newcastle-Upon-Tyne, UK

## Past

Smoking is established as a risk factor for developing both esophageal adenocarcinoma and squamous cell carcinoma.^1^ In addition, it is known to increase perioperative risk. Esophagectomy is associated with high levels of morbidity, and smoking is known to increase these risks.^2^ However, it is not clear what impact smoking status, with regards to being either a current, ex-, and non-smoker, has on perioperative outcomes and long-term survival.

## Present

The present study highlights the very real risk that smoking presents for patients undergoing esophagectomy for cancer.^3^ Complications were higher (73% for smokers versus 62% for non-smokers), as was critical care stay (3 days versus 2 days). This translates to additional costs to healthcare providers. Most importantly, this paper shows the impact on overall survival, with current smokers having median survival of 36 months compared with 48 months for those who have never smoked, although patients who smoked and received neoadjuvant treatment did not have reduced overall survival.

## Future

How smoking affects long-term survival in esophageal cancer patients is still not fully understood. Perhaps the most important question that remains is whether there is a significant benefit for those who smoke at time of diagnosis but then stop at this point. There is an indication that ex-smokers do better than those who continue to smoke, but the data on timing of stopping smoking and how this influences prognosis require further study. There may be further genetic profiling that can help identify patients in whom smoking is more detrimental or negates neoadjuvant benefit.
